# A genome-wide association study of a rage-related misophonia symptom and the genetic link with audiological traits, psychiatric disorders, and personality

**DOI:** 10.3389/fnins.2022.971752

**Published:** 2023-01-24

**Authors:** Dirk J. A. Smit, Melissa Bakker, Abdel Abdellaoui, Alexander E. Hoetink, Nienke Vulink, Damiaan Denys

**Affiliations:** ^1^Department of Psychiatry, Amsterdam University Medical Center, University of Amsterdam, Amsterdam, Netherlands; ^2^Amsterdam Neuroscience, Amsterdam, Netherlands; ^3^Department of Otorhinolaryngology–Head and Neck Surgery, University Medical Center Utrecht, Utrecht University, Utrecht, Netherlands; ^4^University Medical Center Utrecht Brain Center, Utrecht University, Utrecht, Netherlands

**Keywords:** hatred for chewing sounds, psychiatric genomics, psychiatric nosology, genetic correlation, audiology, psychiatry

## Abstract

**Introduction:**

People with misophonia experience strong negative emotional responses to sounds and associated stimuli—mostly human produced—to an extent that it may cause impairment in social functioning. The exact nature of the disorder remains a matter of ongoing research and debate. Here, we investigated the genetic etiology of misophonia to understand contributing genetic factors and shed light on individual differences in characteristics that are related to the disorder.

**Methods:**

For misophonia, we used an unpublished genome-wide association study (GWAS) from genetic service provider 23andMe, Inc., on a self-report item probing a single common misophonic symptom: the occurrence of rage when others produce eating sounds. First, we used gene-based and functional annotation analyses to explore neurobiological determinants of the rage-related misophonia symptom. Next, we calculated genetic correlations (*r*_G_) of this rage-related misophonia symptom GWAS with a wide range of traits and disorders from audiology (tinnitus, hearing performance, and hearing trauma), psychiatry, neurology, and personality traits.

**Results:**

The rage-related misophonia symptom was significantly correlated with tinnitus, major depression disorder (MDD), post-traumatic stress disorder (PTSD), and generalized anxiety disorder (GAD; 0.12 < *r*_G_ < 0.22). Stronger genetic correlations (0.21 < *r*_G_ < 0.42) were observed for two clusters of personality traits: a guilt/neuroticism and an irritability/sensitivity cluster. Our results showed no genetic correlation with attention deficit and hyperactivity disorder, obsessive-compulsive disorder, and psychotic disorders. A negative correlation with autism spectrum disorder (ASD) was found, which may be surprising given the previously reported comorbidities and the sensory sensitivity reported in ASD. Clustering algorithms showed that rage-related misophonia consistently clustered with MDD, generalized anxiety, PTSD, and related personality traits.

**Discussion:**

We conclude that—based on the genetics of a common misophonia symptom—misophonia most strongly clusters with psychiatric disorders and a personality profile consistent with anxiety and PTSD.

## Introduction

Misophonia is a condition in which trigger sounds—such as chewing or breathing—provoke disproportionately strong and involuntary feelings of anger, anxiety, and/or disgust. When severe enough, these emotional responses (or the associated avoidance behavior) may impede family relations and/or work life, resulting in patients seeking help from healthcare professionals. Recently, a consensus panel was unable to converge on a clear nosology for misophonia, and classified it as either a “psychiatric disorder” or the more general “medical disorder” (Swedo et al., 2022). The expert panel also concluded that knowledge on the genetic and neurobiological underpinnings of misophonia are lacking, and that further investigation is needed into the relation of misophonia with other disorders to better characterize misophonia. In addition, such research should also focus on the dependency of misophonia on contextual factors (such as personality) that influence interpretation of misophonic trigger sounds and thus modulate disease etiology (Jastreboff and Jastreboff, 2001a,b, 2015).

Our present research aims to fill in one of the gaps that the expert panel highlighted, namely, the genetic underpinnings of misophonia. Our study is primarily based on the analysis of a Genome-Wide Association Study (GWAS) of a common misophonia symptom, namely, a self-report item on rage induced by chewing-sounds and analyzed by genetic service provider 23andMe, Inc. (San Francisco^1^). Using this symptom as a proxy variable for misophonia, Fayzullina et al. (2015) reported one genetic locus that was significantly associated with the misophonia symptom. This genetic locus, rs2937573, is intronic to the TENM2 gene that plays a role in cell adhesion and is highly expressed in neurons in various stages of brain development. However, a functional annotation of these results has not yet been performed, including the role of TENM2 in hearing and psychological traits as revealed by the GWAS. Our first aim is to perform this analysis, which may provide insights into the neurobiological underpinnings of misophonia.

Our second aim is to determine the association between the genetics of the rage-related misophonia symptom with the genetics of many other traits. It is known that genetic etiology of disorders (including psychiatric, neurological, and many other disorders and traits) show pervasive correlations (Bulik-Sullivan et al., 2015). This overlap shows strong clustering (Lee et al., 2019) across psychiatric disorders, for example, a substantial degree of overlap (genetic correlation *r*_G_ = 0.31) was reported between obsessive-compulsive disorder (OCD) and bipolar disorder (BIP). Moreover, shared genetics extends to substance use disorders (Abdellaoui et al., 2020) and non-psychiatric variables such as socio-economic status, which has important consequences for nosology and identification of contributing factors (Marees et al., 2020b). Inspecting genetic correlations and placing misophonia in a network of disorders and traits will aid its nosology.

We selected a list of 44 traits and disorders for our genetic correlation analysis. Based on the phenotypic comorbidities of misophonia with psychiatric disorders, it seems most likely that misophonia will show significant genetic correlations with major depression, autism spectrum disorder (ASD) and attention deficit and hyperactivity disorder (ADHD) (Jager et al., 2020), possibly also with OCD and Tourette’s syndrome (Webber et al., 2014; Webber and Storch, 2015). In addition, we expect misophonia to correlate with personality dimensions (Jager et al., 2020). Therefore, personality traits will be added to the list of GWAS that may classify misophonia. A second group of disorders and traits comes from the field of audiology. Initially defined as a form of decreased sound tolerance (Jastreboff and Jastreboff, 2001a,^2014^), misophonia may bear relation to audiological disorders and related traits. Finally, we added several traits that putatively bear relation to misophonia. Neurological traits may reflect neuronal excitability; cortical measures of the limbic cortex (viz., mean insula surface area and thickness) (Grasby et al., 2020) were included based on the Autonomous Sensory Meridian Response (ASMR) hypothesis of misophonia (McGeoch and Rouw, 2020). Finally, educational attainment is known to correlate with many psychiatric disorders as well as audiological performance measures, and was added for this reason.

## Materials and methods

### GWAS summary statistics

The source GWASs are studies from 23andMe, UK Biobank and the Psychiatric Genomics Consortium (PGC). Supplementary Table 1 shows an overview of all the disorders and trait GWASs used, their sample sizes, and their source, and specifics on the measurement. All psychiatric disorder GWAS are case-control GWAS from the PGC with clinically ascertained samples.

A case-control GWAS for misophonia based on the proposed clinical criteria does not yet exist, nor was the condition assessed in the UK BioBank. However, Fayzullina et al. (2015) published a GWAS on the self-reported item of “Does the sound of other people chewing fill you with rage?” in 80,607 subjects from the general population, including only subjects who answered yes or no to the question. These results have not been the subject of a peer review process. The prevalence of a positive answer to this question was 22%, which is an overestimation of the clinical prevalence of misophonia, 5–5.9% in Germany, 12.8% in Turkey and 18% in the UK (Kılıç et al., 2021; Jakubovski et al., 2022; Vitoratou et al., 2022). It has been shown that anger is a very common emotional response in 89.5% of misophonia cases and that chewing sounds are a misophonia trigger sound for 95% of cases (Jager et al., 2020), making the single item highly representative of misophonia. All study participants were required to have over 97% European ancestry, as determined by analysis of local ancestry (Durand et al., 2014). The reference population data for ancestry analysis were derived from public datasets (the Human Genome Diversity Project, HapMap, and 1,000 Genomes) and from 23andMe customers who have reported having four grandparents from the same country. At present, the database has the highest power to detect associations in cohorts of European ancestry.

Wherever possible, European ancestry versions of the summary statistics of other traits were selected, since results from other ancestries may bias the results. There was no selection on gender. A total of 43 traits were compared with misophonia in this study. These traits were categorized as Audiological (10, including tinnitus and hearing performance traits), Psychiatric (11) and Personality (15). The remaining 8 traits were added to the category “Other” and included various neurological disorders (Alzheimer’s, Parkinson’s, epilepsy), insula measures (surface area and thickness), and socioeconomic factors Educational Attainment and Townsend Index.

Several UK Biobank trait GWAS (mainly for personality traits) were obtained from the NealeLab GWAS collection web page (NealeLab., 2018).

### GWAS of hearing traits

For several audiological traits a GWAS using the UK Biobank data was performed. To establish whether hearing problems may play a role, a quantitative GWAS was performed on the hearing test results (field 20019 and 20021, “Speech perception threshold” left and right ear using the speech-in-noise test). Additional case-control GWASs were performed for Hearing aid, Hearing problems, and Loud music exposure (fields 3393, 2247, 4836).

For tinnitus (UK Biobank field 4803, “Do you get or have you had noises (such as ringing or buzzing) in your head in one or both ears that lasts for more than 5 min at the time?”), two analyses were performed, one for “Ever Tinnitus” (combining all values from “yes, but not now, but have in the past”). In addition, one GWAS was estimated for “Current Tinnitus” (combining all values from “some of the time” and up, with the “yes, but not now, but have in the past” values removed). The Current Tinnitus GWAS was repeated for subjects with good hearing (below −5.5 dB on the hearing test) to test the genetics of tinnitus without functional hearing loss (Dawes, 2013).

### Genetic annotation

We used the functional mapping and annotation (FUMA) web-application (Watanabe et al., 2017) to perform gene based analysis with MAGMA based on chromosomal position. In addition, we identified expression quantitative trait loci (eQTLs) for different tissues from the GTEx (Lonsdale et al., 2013), BRAINEAC,^2^ and eQTLgen (Võsa et al., 2021) resources. Supplementary Table 2 gives an overview of the tissues selected for the analyses.

We subsequently ran a Transcriptome-Wide Association Study (TWAS) for the GTEx tissues (version 8) using the FUSION software (Gusev et al., 2016). The TWAS resulted in a test for each tissue by gene combination reflecting the genetic association of misophonia with a gene for that particular tissue. Significance levels of these tests were FDR corrected across all tested genes within a tissue. These were further corrected for the multiple tissues: To correct for multiple testing of expression profiles across tissues—which are expected to highly correlate—we estimated the independent degrees of freedom of the cross-tissue imputed expression correlation matrix with spectral decomposition (Nyholt, 2004; Li and Ji, 2005). This number was used as a Bonferroni correction factor. The correlation matrix for input into the spectral decomposition was based on pairwise complete TWAS z-scores.

### Genetic correlations

To calculate the genetic correlation between the set of 44 GWASs, the package GenomicSEM (Genomic structural equation modeling, Grotzinger et al., 2019) was used in R (version 4.0.3, R Core Team, 2022). With this package, pairwise bivariate LD score regression analyses were performed using the recommended settings across all traits (Bulik-Sullivan et al., 2015). Single nucleotide polymorphism (SNP) filtering settings were HWE *p* > 1E-8, MAF > 0.01 insofar available, and were downsampled to HapMap 3 excluding the MHC region for the subsequent genetic correlation calculation (Bulik-Sullivan et al., 2015).

For visualization of the genetic correlations R-package corrplot (version 0.86) was used, using hierarchical clustering to order the traits. Before clustering, we identified traits that were reverse coded—that is, all traits that reflect positive aspects such as friend satisfaction were reversed. This was done by entering the full genetic correlation matrix into an Eigen decomposition and extracting the loadings on the first unrotated principal component. Of traits with substantial negative loadings (below −0.05) all genetic correlations were negated. All *p*-values were corrected for multiple testing using the False Discovery Rate control (Benjamini and Hochberg, 1995).

### Graph clustering

R package iGraph (version 1.2.6, Csardi and Nepusz, 2006) was used to determine the clustering of all traits included in the analysis using the Louvain method (Blondel et al., 2008). The method optimizes the modularity index q, which indexes the relative size of within cluster strength of the within-cluster strengths compared to the between-cluster strength, where strength was defined as the genetic correlation between trait pairs.

To establish the consistency of clustering we used the data provided by GenomicSEM to resample the genetic correlation matrix. GenomicSEM provides variability (standard errors) of the estimates together with the covariation between estimates. This matrix (a 990 × 990 matrix for 44 traits) was used as the “sigma” matrix in the R package mvrnorm, with the estimates (genomicSEM matrix S) themselves as the “mu” parameter. This provided a set of 1,000 samples with the resampled estimates in a single row with the correct mean value, variability, and covariability between the resampled estimates. Each resampling was reordered into a genetic correlation matrix, and reassessed with the Louvain clustering method. Finally, we counted the number of times pairs of traits were grouped in the same cluster to assess the consistency of clustering.

## Results

### Misophonia GWAS and annotation

Supplementary Figure 1 shows the Manhattan plot for the rage-related misophonia symptom GWAS (see also Fayzullina et al., 2015). The top SNP rs2937573 was highly significant (*p* = 2.58 × 10^–43^) and is located near the TENM2 gene (intergenic in build37; intronic in build38). None of the SNPs within the LD block (cutoff *r*^2^ = 0.6) is a known eQTL for a gene, but several deleterious SNPs are present in the region, of which rs2915860, rs7728595, and rs2915858 were present in the main GWAS and significantly associated with misophonia (*p* ≤ 1.28 × 10^–11^; CADD ⇒ 15.36). Remaining SNPs in the LD block with CADD > 12.37 are listed in Supplementary Table 2 (Kircher et al., 2014). Opentargets.org and GWAS catalog lookup of the top SNP and SNPs in LD reported a link with adolescent scoliosis (Liu et al., 2018) and “Time spent watching TV” (UK Biobank item 1080, analysis Neale v2).

A second independent hit (rs7522520, *p* = 3.57 × 10^–8^) was located on chromosome 1 near pseudogene RN7SK. Supplementary Table 3 shows the SNPs in the LD block of rs7522520 (cutoff *r*^2^ = 0.6) as reported in GWAS catalog (MacArthur et al., 2017), which includes a variety of traits in the wellbeing spectrum. One SNP within the LD block is an eQTL for NEGR1 (*Neuronal growth regulator 1*; rs6656687, eQTLgen blood tissue *cis*-eQTL, *p* = 1.94 × 10^–23^). This SNP was not tested in the original GWAS. Supplementary Table 4 shows the deleteriousness (CADD) scores of the SNPs in the LD block. The LD block covered 22 SNPs with CADD score > 12.37; none were significantly associated with misophonia.

FUMA positional gene-based analysis with MAGMA showed one Bonferroni corrected significant gene: TMEM256 (corrected *p* = 0.0257) on chromosome 17.

### Expression analysis

We performed a Transcriptome-Wide Association Study analysis (TWAS) using 10 GTEx brain tissue and 1 whole-blood expression profiles. To correct for multiple testing, we applied the Benjamini-Hochberg false discovery rate (FDR) to adjust for testing across many genes (Benjamini and Hochberg, 1995). Across tissues, we further applied Bonferroni correction to the significance threshold, using the estimated true degrees of freedom using MatSpD (Nyholt, 2004). Since MatSpD estimated *df* = 3.84 as the effective degrees of freedom of the TWAS effects across tissues, alpha = 0.0130 was used as the significance cut-off value. The TWAS revealed that *TFB1M* expression in Hippocampal tissue was the only significant effect (FDR-*p* = 0.0055). *TFB1M* is located on chromosome 6 and encodes for one of several proteins that regulate mtDNA transcription and replication, and is associated with mitochondrial non-syndromic sensorineural deafness, and drug-induced hearing loss (Bykhovskaya et al., 2004; O’Sullivan et al., 2017). *ECE2* gene expression in Putamen was close to significance (FDR-*p* = 0.0168, *not significant*).

### SNP heritability and genetic correlations

#### SNP heritability

**FIGURE 1:**
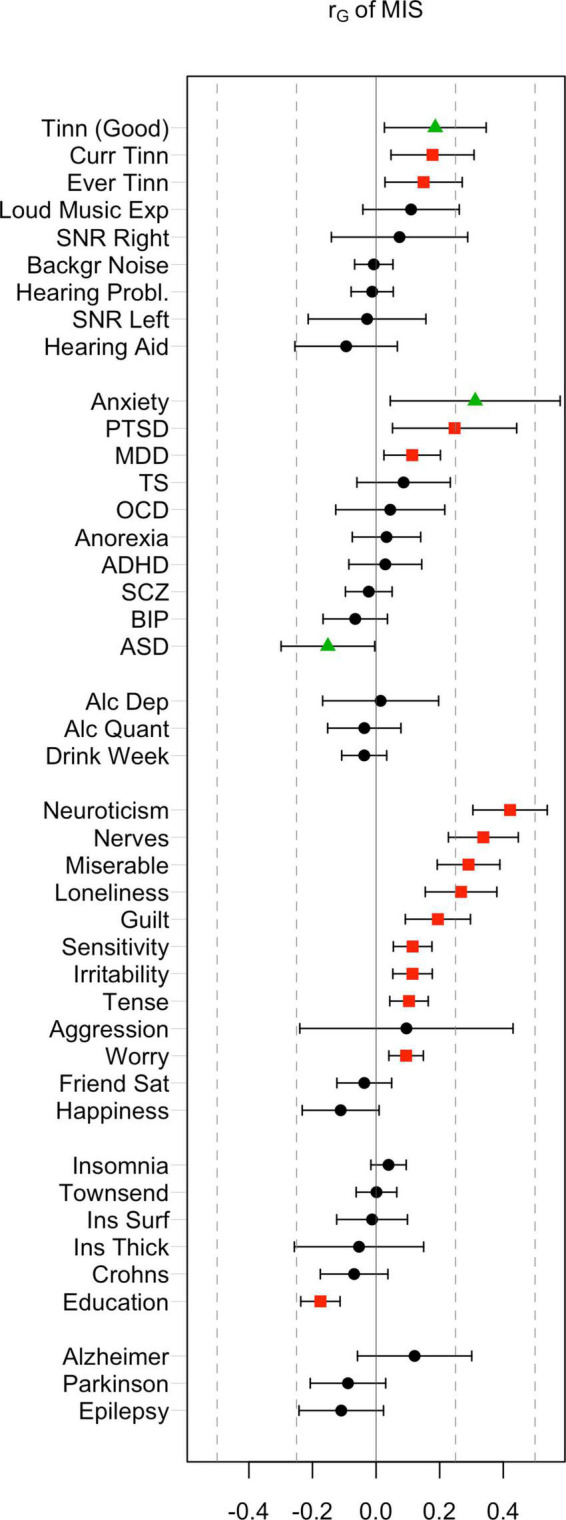
Genetic correlations of misophonia symptoms with a range of behavioral traits and disorders. False discovery rate (FDR)-corrected significant effects (red) were observed in most categories (audiological, psychiatric, personality, and miscellaneous traits). Nominal significance (green triangles) was observed for Anxiety, autism spectrum disorder (ASD), and “Tinnitus in good hearing”. FDR adjusted significant correlations were observed for Current Tinnitus, Ever Tinnitus, post-traumatic stress disorder (PTSD), major depression disorder (MDD), a range of internalizing and externalizing traits, and educational attainment (red).

The rage-related misophonia symptom showed substantial SNP heritability (*h*^2^ = 8.5%, *z* = 10.5, *p* = 9.2 × 10^–26^). The LD-score regression intercept was 1.005 (SE = 0.0088; not significant), indicating excellent control of inflation due to stratification. The genetic correlations of misophonia with the selected traits are shown in Figure 1, clustered by category and ordered by magnitude. The full heatmap of the correlations is provided in the Supplementary Figure 1.

#### Audiology

**FIGURE 2:**
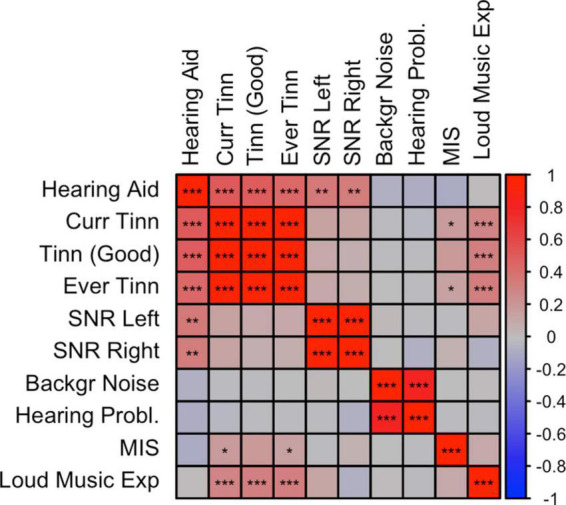
Correlation plot between misophonia and audiological traits. SNP-based genetic correlations were calculated between misophonia and 10 audiology traits using LD-score regression (LDSC). *p < 0.05, **p < 0.01, ***p < 0.001, false discovery rate (FDR) adjusted across all traits combinations. See Supplementary Table 1 for abbreviations.

Rage-related misophonia showed moderate but significant positive correlations in the range from 0.15 to 0.19 with tinnitus (specifically, Ever tinnitus and Current tinnitus). Correlations with other audiology traits were not significant. In contrast with rage-related misophonia, the three tinnitus traits showed clear overlap with hearing trauma variables (current tinnitus: *r*_G_ = 0.51 for loud music exposure; *r*_G_ = 0.29 for hearing aid). This is consistent with the early observations that misophonia is unrelated to hearing performance and/or hearing loss, whereas tinnitus often arises after hearing trauma (Langguth et al., 2013; Jastreboff and Jastreboff, 2014; Moore et al., 2017). Loud music exposure is even likely to have a causal effect on tinnitus (Moore et al., 2017). Like misophonia, tinnitus was unrelated to hearing performance (SNR left or right), which seems at odds with tinnitus’ correlation with hearing loss, but may be due to the fact that most of the SNR trait variation within normal hearing range is unrelated to hearing problems (Figure 2). This is visible as moderate correlations between SNR left and right with hearing aid (*r*_G_ = 0.37 and *r*_G_ = 0.34 for left and right SNR, respectively), and non-significant correlations with hearing problems.

Interestingly, loud music exposure showed a pattern of correlations that was very similar to that of the rage-related misophonia symptom (i.e., moderate correlations with tinnitus variables and low correlations with the remaining traits), however, the direct correlation between loud music exposure and rage-related misophonia was low and not significant. This further suggests that misophonia is not related to hearing problems, but reflects shared etiology with tinnitus *via* other (psychological) traits (Pattyn et al., 2016).

#### Psychiatric liabilities

Consistent with the observed phenotypic correlations (i.e., comorbidities), we observed significant positive correlations between misophonia and MDD (*r*_G_ = 0.11, uncorrected *p* = 0.012, FDR-*p* = 0.045) and PTSD (*r*_G_ = 0.25, uncorrected *p* = 0.013, FDR-*p* = 0.045). The strongest correlation was with anxiety (*r*_G_ = 0.31, uncorrected *p* = 0.022). Surprisingly, there was a (nominally) significant negative correlation between ASD and rage-related misophonia (*r*_G_ = −0.15, uncorrected *p* = 0.044, FDR-*p* > 0.05).

#### Psychological/personality traits

Rage-related misophonia was significantly positively correlated with guilt, loneliness, miserableness, nerves, neuroticism, irritability, sensitivity, tense feelings, and worry. The strongest correlation was with neuroticism (*r*_G_ = 0.42, uncorrected *p* = 2.0 × 10^–12^, FDR-*p* = 7.5 × 10^–11^). There were no negative correlations. A positive correlation with aggression did not reach significance, possibly due to the wide confidence intervals.

**FIGURE 3:**
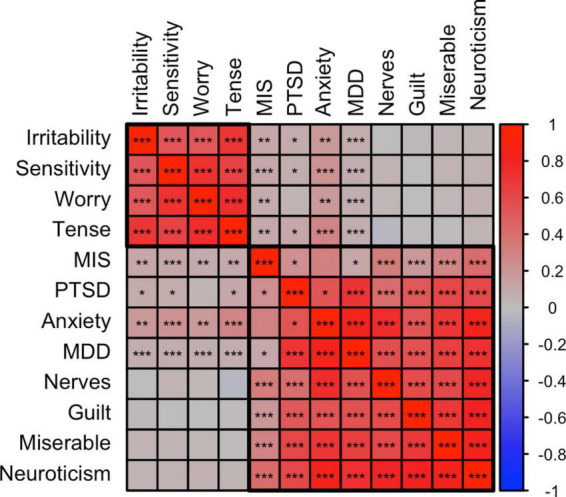
Correlation plot between misophonia, psychiatric, and psychological traits. SNP-based genetic correlations were calculated between misophonia, tinnitus (current), and 12 selected traits shows the membership of two main clusters identified using hierarchical clustering, putatively called the irritability and neuroticism clusters. The neuroticism cluster holds most internalizing traits. The irritability cluster also holds sensitivity, tenseness, and worry. The psychiatric traits are clustered with the neuroticism cluster but all showed significant positive correlations with the irritability cluster. Misophonia closely followed the psychiatric traits. Tinnitus showed a pattern different from misophonia with high correlations with the neuroticism cluster but no significant correlations with the irritability cluster. *p < 0.05, **p < 0.01, ***p < 0.001, false discovery rate (FDR) adjusted across all traits combinations. See Supplementary Table 1 for abbreviations.

Figure 3 shows a correlation plot for a selection of above traits (i.e., significantly correlated to misophonia) plus the MDD, PTSD, and anxiety liability traits that highly correlate with the selected psychological traits. From the correlational pattern two clusters appear, where guilt, nerves, loneliness, miserable, neuroticism correlate highly. Irritability, sensitivity, tense and worry formed a second cluster. Psychiatric disorders (anxiety, PTSD, and MDD) clustered with the neuroticism/guilt cluster, but also showed significant overlap with the irritability/sensitivity cluster. Rage-related misophonia closely followed the pattern of correlations of the psychiatric disorders, as it clusters in the neuroticism/guilt cluster but also shows moderate genetic correlations with the irritability/sensitivity cluster.

#### Remaining disorders and traits

Environmental variables of social and living conditions as well as important cognitive traits are genetically correlated to some psychiatric disorders, including substance use disorders (Abdellaoui et al., 2019, 2020; Marees et al., 2020a). We therefore investigated Townsend index—an index of impoverished living environment—and EA as possible contributing factors for misophonia. These variables are known to have a genetic component (Abdellaoui et al., 2019). Of these, misophonia showed a highly significant negative correlation with educational attainment (*r*_G_ = −0.18, FDR-*p* = 2.8 × 10^–8^). The Townsend index did not show a significant correlation (*r*_G_ = 0.00, *p* > 0.99).

EA is known to have cognitive (IQ) and non-cognitive factors (personality, environment). A recent article parsed genetic variance into these constituent parts, and reported that the genetic correlations between Educational attainment (EA) and psychiatry changed (Demange et al., 2021). Genetic correlations of EA with schizohrenia (SCZ), bipolar disorder, Anorexia Nervosa (AN), and OCD were lower for the cognitive EA variance than for the non-cognitive EA variance, even changing sign for SCZ and bipolar disorder. For misophonia, the reversed pattern was observed. Non-cognitive EA correlated significantly with misophonia (*r*_G_ = −0.162, SE = 0.039, *p* = 3.5 × 10^–5^) and a genetic correlation closer to zero was found for cognitive EA (*r*_G_ = −0.071, SE = 0.039, *p* = 0.07).

There were no significant correlations of misophonia with the neurological disorders and insula measures.

### The genetics of misophonia falls in a personality/psychiatric cluster

**FIGURE 4:**
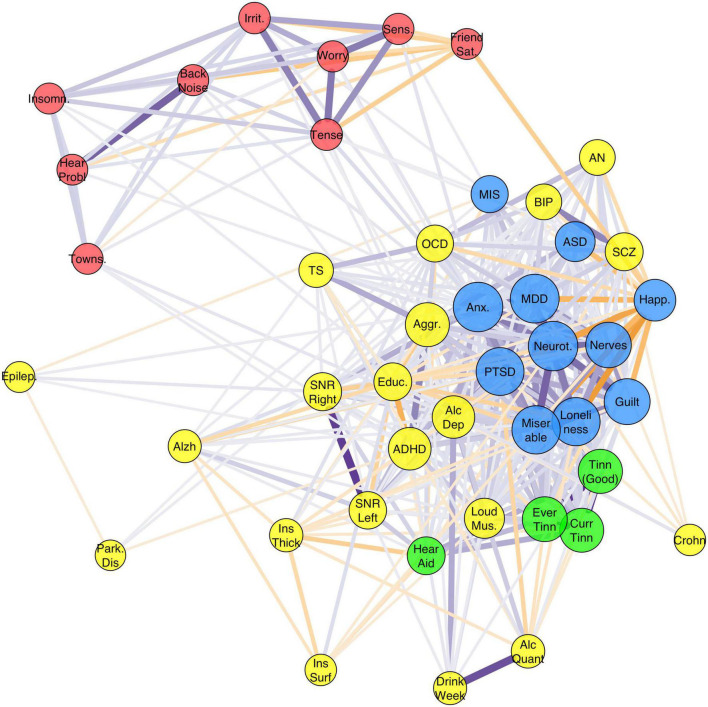
Graph of the full genetic correlation matrix. Vertex colors are based on Louvain clustering algorithm. Per vertex, only the top 10 edges are shown that are over 0.10. Node size is based on the Eigen centrality of the trait calculated from the weighted correlation matrix (absolute values). Misophonia clustered with the Depressive disorders cluster [major depression disorder (MDD), post-traumatic stress disorder (PTSD), and Anxiety] which also holds related personality traits (including neuroticism, guilt, miserableness, loneliness) (blue). Irritability and related traits (worry, sensitivity, tense) cluster with hearing problems (without or with background noise), insomnia, friendship satisfaction, and Townsend index (red). Tinnitus traits clustered with “hearing aid user” (green). The remaining cluster (yellow) holds a variety of traits, which includes neuropsychiatric disorders attention deficit and hyperactivity disorder (ADHD) and autism spectrum disorder (ASD), neurological disorders, substance use disorders, psychotic disorders (schizophrenia, SCZ; bipolar disorder, BIP), obsessive-compulsive-related disorders (OCD; Tourette’s Syndrome, TS; AN), and the insula measures.

The graph based on genetic correlations is shown in Figure 4. Graph clustering showed that rage-related misophonia clusters with psychiatric disorders Generalized Anxiety Disorder, ASD, PTSD, MDD, AN, OCD, schizophrenia, and bipolar disorder, and with psychological traits guilt, miserableness, loneliness, neuroticism, nerves, and happiness. Monte-Carlo resampling of the genetic correlation matrix and recalculating the clustering revealed that the membership of misophonia into this cluster was highly consistent, but this was not the case for all traits and disorders within that cluster. Rage-related misophonia clustered 95% of the samples with PTSD, MDD, guilt, happiness, loneliness, miserableness, nerves, and neuroticism. Cluster concordance was slightly less consistent with anxiety at 87% and with ASD at 61%. Note that the clustering with ASD is based on the consistently negative correlation with misophonia as sign is disregarded in the clustering algorithm. AN, schizophrenia, and bipolar disorder clustered less than 41% of the time with misophonia. Supplementary Figure 3 shows the full cluster concordance matrix.

## Discussion

Our main aim was to investigate the nature of misophonia as a disorder by functionally annotating a GWAS for misophonia to reveal neurobiological function of risk SNPs, and to calculate and cluster genetic correlations between misophonia and a wide range of other disorders and behavioral traits. As the GWAS for misophonia does not yet exist, we used a proxy in the form of a GWAS of a common misophonia symptom assessed in a general population-based sample (23andMe). Two independent locations showed genome-wide significant hits. A highly significant area on chromosome 5 was found, with SNP effects intronic to the teneurin membrane 2 (*TENM2*) gene (for build 38) as well as SNPs in flanking intergenic regions. Drosophila and mouse studies on the teneurin genes influence axonal guidance and synapse formation. In vertebrates, all teneurins (1–4) show a gradient of expression in the thalamus, which putatively guides the axon termination from sensory neurons (Tucker, 2018). Deletion of the region (5q34) reportedly results in mental retardation in humans (Lee et al., 2016; Arya et al., 2020), which would point to an effect this region has on brain development. The results therefore suggest that altered sensory processing based on differential sensori-thalamic neural connectivity could subserve the association of misophonia with TENM2 variants.

However, none of the SNPs in the significant LD block on chromosome 5 are expression QTLs for the TENM2 gene, which may make a pathway through teneurin 2 expression in the brain more problematic. Alternatively, nearby TENM2 on chromosome 5 lie GABA receptor subunit genes (GABRG2, GABRB2, GABRA1), which could also be mediators of the effect as these are well-known genes expressed across the whole brain. GABA receptor genes have been associated with many other disorders (Frajman et al., 2020), so that a mediating role of GABA remains a possibility where it could be hypothesized that functional excitation/inhibition ratios mediate the sensitivity to rage-related misophonia. On the other hand, these GABA receptor genes are mainly reported to be susceptibility genes for epilepsy (Treiman, 2001; Cossette et al., 2002; ILAE Consortium, 2014; Riaz et al., 2021), where no significant association was found between misophonia and epilepsy, which would refute the mediating role for GABA. Future studies may investigate whether teneurin or GABA genes have a role in misophonia etiology, and establish how this could be translated into pharmacological or neuromodulatory treatment.

The second region with significant SNPs was on chromosome 1, and holds expression quantitative trait loci (eQTLs) for neural growth factor 1 (NEGR1), a gene strongly related to various variables related to cognition and socioeconomic status, including cognitive performance and educational attainment (Lee et al., 2018), BMI (Pulit et al., 2019), substance use and protein intake (Liu et al., 2019; Niarchou et al., 2020), and depression symptoms (Baselmans et al., 2019). TWAS implicated TFB1M, a modulator gene for inherited deafness (Bykhovskaya et al., 2004) and associated with intelligence in GWAS (Lee et al., 2018). These functional annotations did not point consistently to specific risk genes or neural mechanisms, even though the GWAS showed significant SNP-based heritability.

Models of misophonia formation and maintenance all have suggested that contextual variables (e.g., personality traits and previous experience) play a role on the positive feedback loop that stepwise increases physiological and behavioral responses to trigger stimuli (Jastreboff and Jastreboff, 2015; Brout et al., 2018). Here, we found that misophonia showed significant genetic correlations with traits and disorders from several categories: misophonia was positively correlated with the assessments of tinnitus (an audiological disorder), with case-control GWAS of PTSD, MDD and generalized anxiety (psychiatric disorders). The strongest correlations were observed with a range of personality traits that broadly fell into two categories, roughly categorized as neuroticism/guilt and irritability/sensitivity trait clusters. In addition, a negative correlation was observed with educational attainment, in line with the functional annotation of the GWAS.

Our findings are largely consistent with the extant literature on comorbid disorders, with some notable deviations. Previous research has reported on comorbidities of misophonia with psychiatric disorders, and on correlations of misophonia with symptoms and personality traits. The reported comorbid disorders that we were able to include in our analyses were PTSD, AN, bulimia nervosa, ADHD, and Autism Spectrum Disorder (ASD) (Schröder et al., 2013; Erfanian et al., 2019). One extensive study of psychiatric comorbidities (Jager et al., 2020) reported no axis I comorbidity in 72% of patients diagnosed with misophonia and no axis II disorders in 59%, and consisted of mood disorders, ADHD, and ASD. Contrary to the earlier reports (Schröder et al., 2013; Erfanian et al., 2019) no comorbid PTSD was found. For axis II, comorbid obsessive-compulsive personality and borderline personality disorders were observed. Obsessive-compulsive personality *traits*, however, were found in 26% of the patients, and clinical levels of perfectionism were found in over 66% of misophonia sufferers. Our results, however, did not show a significant correlation with OCD. A recent study into psychiatric comorbidities of misophonia (Rosenthal et al., 2022) was performed in a community sample of cases using the structured clinical interview of the DSM-V (SCID-5). Here, high rates of lifetime and current social anxiety, generalized anxiety, and specific phobias were reported (with a 73% rate for any anxiety disorder). In addition, the study reported high rates of lifetime MDD and persistent depressive disorders (with 61% total for any mood disorder), which is consistent with our genetic findings. However, they also found elevated levels of lifetime OCD (13.5%), ADHD (17.9%) and Alcohol Use Disorder (20.8%). These comorbidities were not reflected in our genetic analyses. A recent extensive study (Siepsiak et al., 2022) drew a similar picture of heightened incidence of various anxiety disorders, PTSD, AN and OCD in a Polish community sample. In addition, misophonia cases showed increased incidence of a major depressive episode, while MDD was not reported as a comorbid disorder. OCD, AN and other disorders were only slightly elevated in misophonia cases, and may not have been significant.

The genetic correlation of misophonia with tinnitus is consistent with the disorder first described in the literature as similar to but different from tinnitus, hyperacusis, and phonophobia (Jastreboff and Jastreboff, 2001a,b). However, misophonia did not correlate with any of the hearing performance or hearing loss traits (Jastreboff and Jastreboff, 2014, 2015; Jager et al., 2020), in stark contrast to tinnitus that showed significant correlations with Loud Music Exposure and Hearing Aid. Tinnitus did not correlate strongly with SNR, which may be considered surprising as tinnitus is often associated with hearing loss (Langguth et al., 2013). It is also consistent with findings that people with tinnitus but normal hearing thresholds may have hair cell damage or otherwise affected cochlear regions, suggesting that hearing loss does not necessarily lead to loss of hearing performance that can be detected with the hearing test of the UK BioBank (Weisz et al., 2006; Job et al., 2007; Langguth et al., 2013). Although the results indicate that misophonia is not related to hearing loss (or increased sensitivity), methodological issues remain. The triple digit test is not the gold standard, nor the most sensitive measure of hearing sensitivity. More sensitive measures of hearing sensitivity, such as measurement of hearing threshold or measurement of speech-in-noise perception with CVC words, may reveal such a relationship. We note that no GWAS is available of hyperacusis or phonophobia, therefore, our results do not preclude the possibility that other sensory problems than hearing loss play a role in misophonia.

Contrary to the expectations from comorbidity analyses (Jager et al., 2020; Williams et al., 2021), a negative correlation with ASD was observed. The emotional response that defines misophonia may also be found in patients with ASD (Jastreboff and Jastreboff, 2014; Williams et al., 2021), but this is not reflected in an overlap in genomic variation. It has been noted that despite the decreased sound tolerance observed often in ASD, misophonia sufferers with comorbid ASD are a minority of the misophonia cases (3% in Jager et al., 2020), with ASD cases more frequently forming hyperacusis (Williams et al., 2021). Nevertheless, over 25% of children with hyperacusis—most of which had clinical ASD—indicated having misophonia symptoms (Amir et al., 2018). Our results suggest that misophonia and ASD are relatively independent disorders with regard to genomic variation, the small protective effect suggestively being mediated by the positive correlation of ASD with cognition [*r*_G_(ASD,EA) = 0.21]. It raises the possibility that other forms of misophonia exist, one that is mostly driven by conditioning of anger or other negative emotionality to specific trigger sounds moderated by personality traits; the second forming a smaller subgroup that is driven to a greater extent by decreased sound tolerance (Williams et al., 2021), which was not picked up by the current misophonia GWAS in a population-based sample. Future studies may investigate the specifics of the relation between ASD and misophonia.

Another result that could be considered unexpected is that the positive correlation with aggression was not significant, even though anger and aggressive thoughts are often reported symptoms of misophonia (Bruxner, 2016; Dozier and Morrison, 2017; Jager et al., 2020; Norris et al., 2022). It has been argued, however, that misophonia is based on the feelings of guilt about the evoked irritation and anger rather than behavioral expressions of anger itself that causes the distress (Jager et al., 2020) making the disorder more compulsive and internalizing than impulsive and externalizing in character (Eijsker et al., 2020). Others have shown that a fear of uncontrolled emotional response is an important factor in misophonia, while externalizing thoughts are still important (i.e., blaming others for being the source of the trigger sound) (Vitoratou et al., 2022). It should be noted, however, that the GWAS for aggression was relatively small, and future updates of the aggression GWAS may show a significant positive correlation. The GWAS for another impulsive disorder (viz., ADHD) had, however, ample power. The lack of genetic correlation with ADHD—and lack of clustering of misophonia with in the ADHD/aggression cluster—provides further evidence for the disorder not belonging in the impulsive disorders cluster.

Almost no correlation was found with Anorexia (*r*_G_ = 0.03, *p* > 0.05), in contrast to previous studies into phenotypic comorbidities of misophonia (Kluckow et al., 2014; Erfanian et al., 2019). Lastly, there was barely any correlation with OCD (*r*_G_ = 0.04, *p* > 0.05), even though previous studies did report a link (Webber and Storch, 2015; Wu et al., 2014; Erfanian et al., 2018). The explanation for this could be found in the distinction between OCD and obsessive-compulsive personality disorder (OCPD). For example, Jager et al. (2020) found OCD and OCPD to be comorbid with misophonia, but at a different level: 2.8% of the patients had comorbidity with OCD, while 26% had comorbid symptoms of OCPD. This former prevalence is only slightly higher than population prevalence for OCD (1.6%; den Braber et al., 2016), but the latter is a substantial increase for OCPD [7.8% in the US; (Grant et al., 2004)]. However, to date no GWAS of OCPD has been performed, precluding its use in the current analysis. Nevertheless, the finding that misophonia clusters with psychiatric disorders and related personality dimensions seems to support it either as a highly specific variant of OCPD or a separate personality disorder with strong comorbidity.

Finally, a surprising result was the negative genetic correlation with Educational Attainment, which was significant after correction (*r*_G_ = −0.18, *p* = 3.1 × 10^–7^). Educational Attainment is well-known to correlate with many psychiatric disorders, in part as a non-cognitive indicator of environment or SES (Abdellaoui et al., 2019; Demange et al., 2021). The pattern of genetic correlations of misophonia closely mimicked that of MDD, that also showed a small but significant negative correlation with educational attainment. In addition, MDD showed a stronger association with the non-cognitive variance in educational attainment than the cognitive variance (Demange et al., 2021; Figure 4). Again, this resonates well with the interpretation of non-cognitive variance in educational attainment as personality related. Another indicator for socioeconomic status—the Townsend Index—did not correlate with misophonia. We therefore do not expect that socioeconomic environmental factors play a substantial role in misophonia symptoms.

The results of the graph clustering concurred with the hierarchical clustering observed in Supplementary Figure 2, placing it in a cluster with MDD, PTSD, Guilt, Nerves, Happiness, Loneliness, Neuroticism, and Anxiety. Monte-Carlo resampling of the genetic correlation matrix showed that this clustering was highly consistent (>95%). This leads us to the conclusion that misophonia may be classified as a psychiatric disorder related to MDD and PTSD, with contributing personality dimensions in the guilt/neuroticism spectrum. In addition, personality dimensions from the irritability cluster contribute to the disorder to a lesser degree. Consistent with the genetic correlation analyses—but inconsistent with clinical observations (Jager et al., 2020)—impulsive disorders/traits like aggression and ADHD do not cluster with misophonia.

The main limitation of the current study is the fact that the GWAS of misophonia was based on a self-reported symptom of misophonia rather than a case-control study of misophonia. In addition, the GWAS sampled a common symptom of misophonia with anger as a primary response (Jager et al., 2020; Norris et al., 2022). Most research groups seem to agree that misophonia is independent of the overt expression of anger as a primary emotional response (Jager et al., 2020; Norris et al., 2022; Siepsiak et al., 2022; Vitoratou et al., 2022). However, many groups also include a wider variety of primary emotional responses than irritability or anger (anxiety, panic) (Vitoratou et al., 2022). Although it is currently unknown whether these other emotional misophonia responses would reveal a similar pattern of genetic correlations, it may be argued that the genetic makeup—and therefore its underlying neurobiological determinants—should not vary too much with emotional response as this may point to different underlying neurobiological determinants that contribute to misophonia formation and maintenance. With the aid of GWAS of multiple symptoms, future studies may investigate whether different symptoms in misophonia result in different genetics.

A further limitation is the availability of published GWAS, which is currently lacking in analyses of psychiatric personality disorders such as OCPD, and of (cognitive) symptoms of inflexibility and perfectionism as part of the part of the OCPD spectrum. In addition, GWAS of the disorders phonophobia and hyperacusis are lacking. As more GWAS become available, future studies could investigate whether individual differences in sympathetic nervous system functioning and functional connectivity between auditory and limbic systems are related to misophonia. Moreover, it could be established whether the overlap is similarly responsible for the genetic overlap with PTSD.

On a methodological note, participation in the UK Biobank and 23andMe participation may come with a participation bias, likely selecting on higher educational attainment. This selection bias may limit the generalizability of the results to the more educated part of the population. In addition, the study by 23andMe was restricted to EU ancestry. This limited ancestry limits the generalizability of the results, however, restriction the analyses to EU was required for genetic correlation analyses. Explicitly limiting ancestry opens up the possibility to investigate the transferability of misophonia genetics to other populations, for example, by polygenic scoring in misophonia case-control designs in populations of African (-American) or Asian descent. These biases may result in spurious associations, known as collider bias. These biases are inherent to most studies, and could be addressed in the future by matching bloodspot genetic data to hospital records (see e.g., Pirastu et al., 2021).

To summarize, our results showed significant effects of several SNPs on a typical misophonia symptom, the hatred for chewing sounds. The TENM2, TMEM256, NEGR1, and TFB1M genes are candidates for mediating the effects, as well as GABA genes that are located near the TENM2 gene on chromosome 5. Genetic correlation analysis suggested that misophonia is not merely a sensory disorder related to sensory trauma or hearing loss, although it does have shared genetic etiology with tinnitus. Misophonia is related to personality traits in the neuroticism/guilt cluster, and, to a lesser degree, to the irritability/sensitivity cluster. Finally, misophonia shares genetic etiology with PTSD, MDD, and anxiety disorders. Our conclusion may aid DSM classification and could suggest that different therapy approaches are possible for patients classified on the contributing personality dimensions. To further strengthen this conclusion, more research is needed, and the number of GWAS needs to be extended.

## Data availability statement

The original contributions presented in this study are included in the article/Supplementary material, further inquiries can be directed to the corresponding author.

## Ethics statement

This study was secondary analysis of published GWAS results that were approved by their respective ethics committees. Written informed consent to participate in this study was provided by the participants or their legal guardian/next of kin.

## Author contributions

DS, MB, and AA performed the analyses and provided code for the analyses. DS and MB wrote the manuscript. AH, NV, and DD reviewed and revised the manuscript. All authors contributed to the article and approved the submitted version.

## Footnotes

1
http://www.23andme.com

2
http://www.braineac.org
