# Supplementary material for: A genome-wide association study of a rage-related misophonia symptom and the genetic link with audiological traits, psychiatric disorders, and personality
Source: Front Neurosci. 2023 Jan 24;16:971752. doi: 10.3389/fnins.2022.971752 (PMC9902885; doi:10.3389/fnins.2022.971752)
Supplement: Supplementary file 2 [file Data_Sheet_2.docx]

**
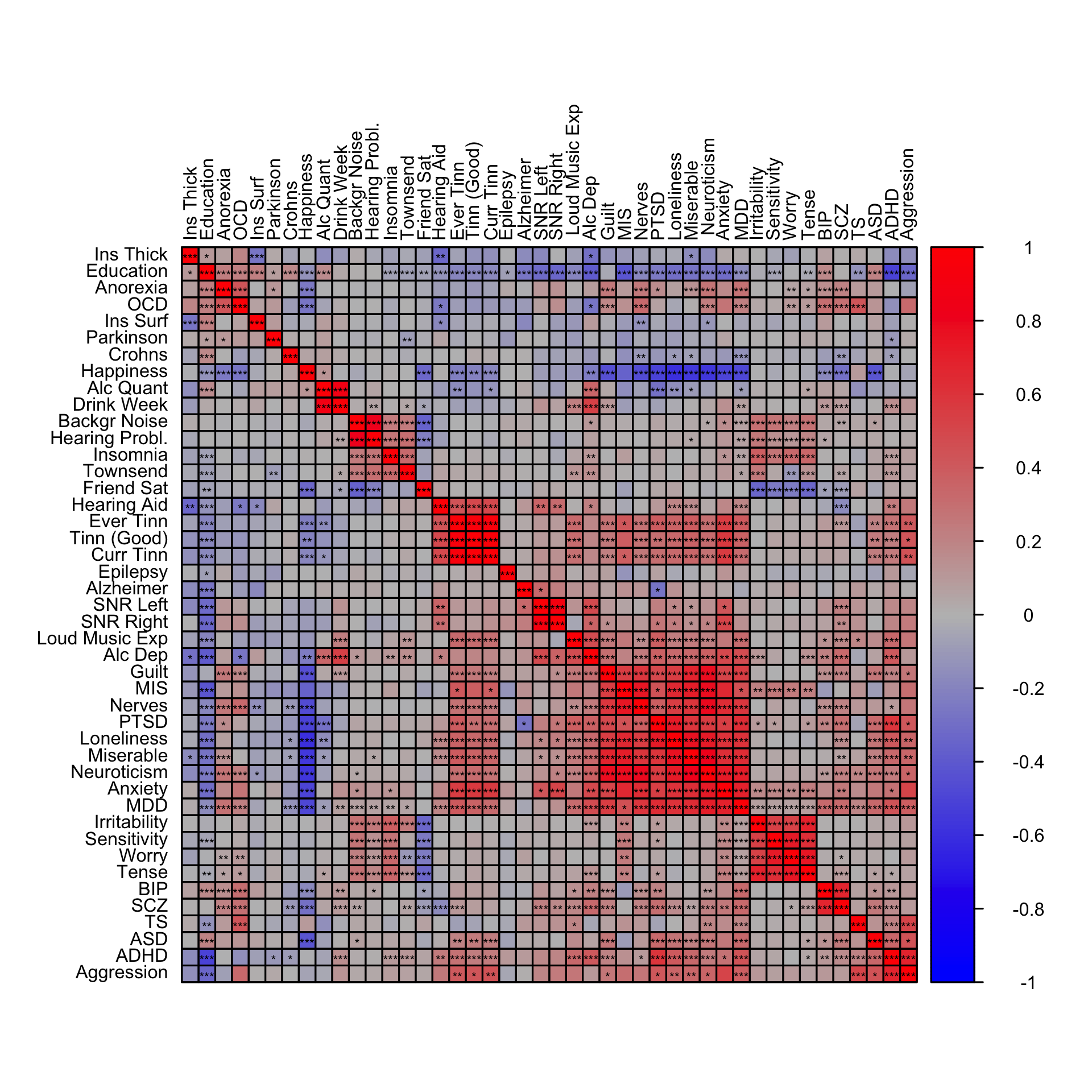
**

**Figure S1.** The full genetic correlation heatmap ordered using hierarchical clustering. Significance was based on FDR-corrected p-values across the full lower diagonal of the matrix. *p<0.05, **p<0.01, ***p<0.001.


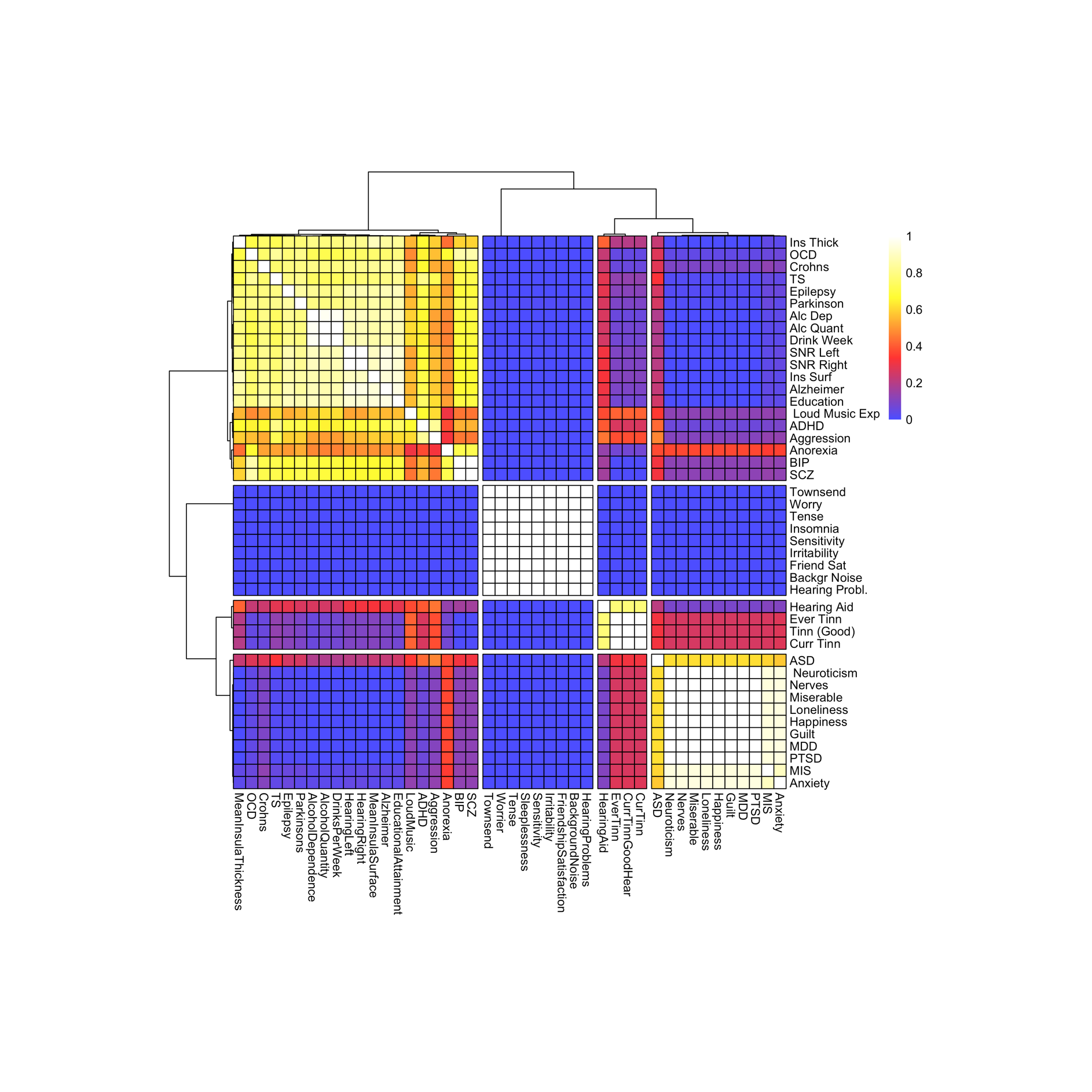


**Figure S2.** Concordance of the graph clustering after Monte-Carlo resampling of the genetic correlation matrix. Each resampling of the genetic correlation matrix is entered into the Louvain clustering method and the cluster membership noted. Then, clustering concordance (i.e., the proportion of times two traits were clustered together) was calculated between all possible pairs in the range (0, 1) for all four cluster solutions (67%). For further details on the resampling of the genetic correlation matrix see methods. The heatmap reveals that misophonia (MIS) is consistently clustered with Guilt, Neuroticism, Loneliness, PTSD, MDD, Happiness, Nerves, and Anxiety.
